# Establishment and Use of Polio Communication Network in Response to Polio in Outbreak Countries of the Horn of Africa: 2013–2014

**DOI:** 10.29245/2578-3009/2021/S2.1117

**Published:** 2021-04-12

**Authors:** Rustam Hydarav, Obianuju Igweonu, Saumya Anand, Mwakisha Jemimah, Almaz Merdekios, Leila Abrar, Joseph Okeibunor, Sam Okiror

**Affiliations:** 1 UNICEF, Nairobi Kenya; 2 University of Nigeria, Nsukka; 3 WHO, Nairobi, Kenya; 4 WHO AFRO, Brazzaville, Congo; 5 WHO Horn of Africa Coordination Office (HOA), Nairobi KENYA

**Keywords:** Polio communication networks, Poliovirus, Outbreak, Response, Horn of Africa

## Abstract

**Background:**

Between 2013 and 2014, the Horn of Africa countries experienced a severe and prolonged outbreak of polio viruses. It started in one district in Somalia but quickly became a national and even international disaster, crossing international boundaries into Kenya and Ethiopia. This paper documents experiences in the establishment and contributions of the Polio Communication Network (PCN) to the polio outbreak response in the outbreak countries of Somalia, Kenya and Ethiopia from 2013 to 2015.

**Process:**

The establishment of the PCN network of partnerships and technical assistance was designed to implement a strategic communication response. Various strategies were used to establish the PCN. Some of these strategies included partnerships with faith-based organizations; involvement of local leaders in microplanning; social mobilization committees and research, monitoring, evaluation and documentation structures.

**Major Outcomes:**

PCN contributions through sustained high levels of community awareness of polio rounds were demonstrated. The contributions of the context-sensitive approaches included significant gains in reaching traditionally missed, hard-to-reach, pastoral communities with polio information, improved communication capacity, and successful closure of the outbreak within the expected timeline. This PCN experience provides important communication lessons relevant to polio eradication and other public health programmes. The focus on building capacity in areas such as monitoring, and data collection generated social data that led to the communication approaches making a significant impact. PCN contributed to a better understanding of the behavioral and environmental factors affecting the demand for, and uptake of, health services in the HoA which can be extended to most of the countries in the HoA with the same demographic and epidemiological realities.

**Conclusion:**

The use of the PCN helped bring the 2013-2014 polio outbreak under control and illustrates how the PCN can help drive progress towards the realization of the agenda of the universal health coverage and vision 2030 agenda in the African Region and elsewhere.

## Introduction

Between 2013 and 2014, the Horn of African (HoA), comprising of ten contiguous countries in the WHO African Region and the WHO Eastern and Mediterranean Region experienced one of the worst poliovirus outbreaks. The outbreak happened at a time the Global Polio Eradication Initiative (GPEI) was set to complete the work of eradicating polio globally^1–7^. The worst hit countries among the ten countries in the HoA were Somalia, Kenya and Ethiopia. They were hit by a related wild polio virus (WPV), while South Sudan was hit with a circulating Vaccine Derived Polio Virus type 2 (cVDPV2) unrelated to the WPV in the other countries.

The 2013 outbreak started in Somalia, when a 32-month-old girl was identified as a case of WPVl in the Mogadishu area (Hamar Jajab district); the date of paralysis onset was 18 April 2013. The outbreak rapidly expanded to all of Mogadishu’s 16 districts, with secondary spread to other areas of South and Central Somalia. By 31 December 2013, 189 cases of WPV1 were notified from 46 of 110 districts of the country, mostly from the security compromised areas of South and Central Somalia^8^. Worse still, the same virus moved swiftly across international boundaries to Kenya within 10 days of its detection in Somalia. This was precisely by the 30th of April 2013 and finally to Ethiopia by 10th July 2013.

This was the first outbreak in Somalia since 2007 and in Kenya since 2011^8,9^ but the transmission time was unprecedented. By 1 July 2013, 25 cases had been reported from Somalia (primarily from Banadir region) and six from Kenya (Dadaab in north-eastern Kenya) by 14 August 2013. With this epidemiological trend, Somalia became reputed for having the worst ever outbreak reported globally in a non-endemic country with 105 cases confirmed. In the same period, close to 10 cases of wild polio were confirmed in Kenya while six cases were confirmed in Ethiopia.

This series of sustained outbreaks in the Horn of Africa threatened the then newly endorsed Polio Eradication and Endgame Strategic Plan 2013-2018 objective of stopping all polio transmission globally by the end of 2014^10^. This reality prompted the Horn of Africa Technical Advisory Group (TAG) to call for intensification of efforts to keep the virus permanently out of the region to complete the job of eradicating polio^11^. The GPEI team in the Horn of Africa responded to the outbreak with an unusual resolve to stop the transmission of polioviruses in this resource poor region^8,12^. Various strategies were developed, tested and deployed to interrupt polio transmission, which at the time was rapidly spreading in the Region. In relatively little time, the polio outbreak began to decline and by the end of 2013, the total number of polio cases stood at 203 (183 from Somalia, 14 from Kenya and six from Ethiopia). By 18 June 2014, the total number of cases in the region was 219 (195 from Somalia, 14 from Kenya and 10 from Ethiopia). However, two new cases of cVDPV2 were reported in the Rubkona district of Unity province in South Sudan in the week of 4 November 2014^8^. Communication and social mobilization played a critical role in reaching communities and fostering polio vaccine acceptance and demand^13,14^.

In its August 2014 meeting, the HoA Polio Technical (TAG) Advisory Group noted three domains of weaknesses. These were weak social mobilization (e.g., poor training tools, supervision and management in the field and team deployment – microplanning); poor outreach to pastoralists (e.g., where to find them, how to build trust with them and reach them with information); and lack of systematic use of data for planning (e.g., timely availability of data, disaggregated analysis for decision making at district level). The TAG also identified possible barriers to each of these domains. For instance, with respect to social mobilization, the listed barriers included workforce turnover, lack of focus on implementation of social mobilization, and weak coordination with operational components in the field. On reaching out to pastoralists, the TAG listed the possible barriers included lack of a focused and systematic approach to issue, convergence with other programmes (eg Polio+) and lack of flexibility in operationalization. Finally, on the failure to systematically use data for planning, the possible barriers included low capacity at district level, short interval immunization rounds and a paradigm shift with respect to answering the “so what?” questions.

The Horn of Africa polio response team thus established a polio communication network (PCN) to address these issues of polio communication and social mobilization. This paper describes the establishment and use of the PCN in interrupting poliovirus transmission in the Horn of Africa.

## Processes

### Establishing the PCN

The PCN is a network of partnerships and technical assistance designed to implement a strategic communication response to the polio outbreak^13^. UNICEF, one of the key partners in the outbreak response led the process of establishing the network in polio outbreak countries with the objective of increasing positive polio knowledge and behavioral outcomes in communities. Based on global experience, as refusal of polio vaccination has been one of the barriers to global polio eradication^15^. The PCN built communication capacity and commitment at local levels, engaged specialized partnerships for social mobilization (such as local, influential leaders and community groups), and used data for action. It also identified, addressed, and preempted refusals to vaccinate, no matter how small the number, as a matter of urgency. The PCN was designed to meet local communication needs with local solutions^13^.

The PCN worked with the different local structures including regional communication coordinators at the different administrative levels in the outbreak countries. It also partnered with relevant religious organs and bodies. In each case UNICEF staff and Ministry of Health experts at the highest levels led the strategic response. They were supported by local coordinators recruited based on their expertise in clinical or public health to support planning, capacity building, advocacy, social mobilization, monitoring, and evaluation. They were provided vehicles to enable wide geographic reach of support, and quarterly reviews took place to monitor performance and build capacity.

### Partnership with the faith-based organizations

Given the influential role of these faith-based organizations (FBOs) in opinion and behavior formation as well as their capacity to reach the people in great numbers, including pastoralists, relevant FBOs were engaged to partner with UNICEF on the project. The main objective of this partnership was enhancement of community awareness of, demand for, and uptake of polio vaccination among others, while building the capacities of the FBO leaders using participatory and sustainable mechanisms. The VFBOs were nongovernmental, civil-society organization comprising a widespread network. The FBOs were led by a national leadership, with representatives at every administrative level.

Standard messages on polio and child survival were developed and reinforced with religious teachings from the Quran or the Bible, in the different outbreak countries. Cascaded trainings were conducted on programme interventions and messages. Representatives of the FBOs participated in the planning, implementation, and evaluation of the polio supplementary immunization activities (SIAs) and developed and utilized SIA monitoring formats to report numbers of missed children, identified and resolved cases of refusals, and mobilization activities conducted including audiences or individuals reached.

### Involvement of Local Leaders in Microplanning

In all the cases, the PCN employed the bottom-up development planning models^16,17^. To facilitate ownership, local innovation, and commitment, the PCN used this development model for microplanning, which was considered an important step in planning quality polio SIAs to determine accurate target population numbers, locations, and community characteristics to better reach missed, hard-to-reach, and mobile populations^13^.

### Social Mobilization Committees

Social mobilization committees (SMCs) were formed at different administrative levels to mobilize communities on a variety of issues. SMCs were commonly comprised of diverse membership of locally influential persons such as religious leaders, administrators, health personnel, and women leaders. Functionality of SMCs varied across the outbreak countries. The PCN regularly monitored the functionality of the SMCs, revitalized dormant SMCs, and/ or supported establishment of new SMCs where none existed. SMCs were supported with training and capacity building, and SMC engagement in health and other topics were monitored.

### Research, Monitoring, Evaluation, and Documentation

The use of data for action, for polio communication, and social mobilization has long been documented and made a GPEI priority^18^. Research, monitoring, and evaluation data were used to inform and improve communication interventions during the polio outbreak response in the outbreak countries of the HoA. Some of the data included SIA data, regular reports, and special research and assessments. SIA data, including community communication checklist were used intra- and post-SIA to measure indicators for polio and routine immunization; WHO monitoring data were collected as part of the independent monitoring (IM) process; and a house-to-house SIA survey was conducted by the polio programme and partners. The IM process collected information on multiple indicators on campaign performance including communication-related indicators (such as the source of information about the SIA; awareness levels; and reasons for noncompliance). In the Somali Region, IM was conducted during the last 2 days of each campaign and for 2 days after the campaign was completed.

### Zero-Dose Case Studies

In partnership with Jigjiga University, the Somali Regional Health Bureau and UNICEF conducted a qualitative case study of polio “zero-dose” children in February 2014 in the region. The study aimed to uncover why children were missing out on polio vaccination despite intensive SIA efforts. Furthermore, the study aimed to document the sociocultural, religious, behavioral, service-related, and other factors that could influence polio vaccination in pastoralist communities to further guide communication interventions implemented by the PCN. The study included in-depth interviews with family members of 14 zero-dose children and 14 case controls, along with interviews and discussions with service providers, community leaders, and others.

## Major Outcomes

### Communication networks strengthened in Somalia

One hundred percent of managers and trainers were trained using the Communication for Development (C4D) training of trainers’ tools. Fifty six percent of the social mobilizers were trained on infection prevention and control (IPC) in 2014 while the 44% or 1484 were trained by the end of February 2015. The trainees included 21 regional and 127 district communication managers. The PCN supervised the DFAs before any campaigns were undertaken and develop clear movement and deployment plans as well as joint microplanning including social maps. Over three thousand (3,323) trained social mobilizers were equipped with flipcharts and visual aids that included topics on polio, expanded programme on immunization (EPI), water, sanitation and hygiene (WASH).

### Sources of information and campaign awareness identified in Somalia

The PCN increased levels of awareness of polio campaign in Somalia. The results showed that there was a 89% level of awareness. The commonest sources of information, was television news with 38%. Health workers constituted the second most popular source of information with 26% while social mobilizers using megaphone reached 16% with polio campaign information. The remaining 4% got their information from advocacy meetings (2%) and Mosques announcements (2%).

### Engaging supreme council islamic affairs and Zonal communication committee (ZCC) in Somali Region of Ethiopia

Ninety-eight new social mobilization committees were established and trained and 360 social mobilization committees were retrained at the Kebele levels. Sixty percent of the Kebeles in the Somali Region had dedicated social mobilization committees and 17,300 volunteers. A total of 1,200 Sheiks in 4,567 Mosques in 30,060 Duqsies were equipped with polio flip charts, EPI speaking book on Somali and flash drive including EPI social behavior film and audio films. Other work tools included job aid on routine immunization and toolkit bags for health extension workers. New settlements and institutions were successfully mapped. These included 1620 pastoral habitations, 390 schools and 86 religious institutions. Also mapped were 190 markets, 123 water points, 29 veterinarian health posts and 15 food distribution points. With these over 50,000 persons were reached through ZCC; 601,201 were reach through Duqsies and 800.650 were estimated to have been reached through Mosques.

### Applied communication research in Ethiopia and Kenya

Anthropology of nomadic pastoralism was undertaken in the HoA. The studies explored the social dynamics, health seeking behaviours, gender roles, decision making mechanisms and communication channels among the nomadic pastoralists. The studies identified over 435,000 children who were refugees in the HoA and over one million who were internally displaced in Somalia alone. The Dadaab refugee camp in Kenya had an estimated population of 500,000 people and was considered the largest refugee camp in the world. Several factors responsible for the movement of the migrant population were identified to include push factors (asylum seekers and refugees, internally displaced and stateless people); pull factors for the economic and social migrants; and periodic multifactorial nomadic pastoralists such as exclusive, trans-human and agro-pastoralists. Their main routes were through Yemen, South Africa, Ethiopia and Sudan seeking water and field resources. At other times the reasons for pastoralist movement is clan dynamics and displacement. The major pastoralist clusters in the HoA were also identified as Karamajong (Uganda, Kenya and South Sudan); Boran (Kenya and Ethiopia) Somali (Somalia, Ethiopia and Kenya); and Maasai (Kenya and Tanzania). Furthermore, the PCN provided direct country support to Kenya on stakeholder and clan mapping, geofocus on Mandera Masabit, Garissa, Isiolo and Wafir among others. In Ethiopia similar support was given for outreach to pastoralist communities, integration of polio with other child survival services.

### Pastoralist tracking in Somalia and Ethiopia

All sites where nomads and migrants can set up their tents – “Dera” were listed out. This involved the careful assignment of supervisors/vaccinators, partners and social mobilization networks to cover the populations of fishermen, nomadic deras, construction sites workers among others. Networks of informants were also created near all sites and villages/wards. These networks were periodically called to ask about incoming people and inform the social mobilization networks about incoming migrants/ nomads. There was also cross notification of listed nomads across districts over phone, short messages services (sms) and email on incoming and outgoing nomads. In Ethiopia the mapping resulted in an increase from 240 to 1,620 new pastoralist settlements identified.

### Puntland: Leveraging Food & Agricultural Organization (FAO) Partnership with Pastoralists

The animal vaccination programme had an existing and trustworthy relationship with over 2,000 pastoralist clan group in Somalia. Trust was key, thus the Somalia DSMCs/ Ministry of Health staff implemented communication protocols for clan elders. The PCN also engaged cattle market broker and also held sessions at water points. Over 26,000 pastoralist parents were reached with integrated services and health education sessions on polio, measles, vitamin A supplementation and oral rehydration solutions.

### Pastoralist focused communication approaches

These were not necessarily posters. Among the pastoralist/Somali cluster it was very much verbal. It used social mobilization flipcharts/session, IPC skills training, which were critical for social mobilizers using clan elders/ Kebele leaders as entry points. There was also BBC short wave broadcasts as well as interactive education through mobile teams/vans and branded animal health products/ materials.

### Pastoralist primary mobile schools in Turkana

This was a scale up of the “Mtoto Kwa Mtoto” (Child to Child) approach. Sixty one primary mobile schools/ head teachers were engaged in the process and over 3,772 pastoralist students were identified as missed children. The interventions included the orientation of teachers on health education and student outreach. It also used the adoption of 20 children in the community to be followed up during the campaigns. This resulted in the decline in missed children from 1.2% to 0.25% in November 2014. There was also an increase in awareness from 86.69% in June to 87.84 % in November 2014, though this might not be statistically significant. Furthermore, the number of zero dose children (in new settlements) decreased from 760 in June to 570 in November 2014.

## Discussion

This paper documented the process of establishing the PCN and explored its contribution to interruption of the polio outbreak in the HoA. It showed the contribution of the PCN to increased SIA coverage and reduction in the proportion of missed children, and significant advances in the quality of communication efforts. Consequently, the outcomes were captured under three main domains, namely: contribution to increased awareness coverage; an established base of knowledge, human resource capacity, and technical expertise for improved child health.

With respect to contribution to sustained high levels of community and parents’ awareness about polio SIAs, it showed that awareness, which was one of the main communication indicators measured within the GPEI, rose significantly. More parents and communities were made to know about planned SIAs prior to the campaign rounds and more children were vaccinated. The IASC partnership made a major contribution by disseminating credible polio and child survival messages to large numbers of people through a variety of channels. For instance, prior to the maturation of the partnership there were gender disparities in the coverage with messages about SIAs. Fewer females than their male counterparts were reached with messages and this was quickly addressed with special strategies implemented to better reach women^13^. Mothers are producers of household health, especially in the case of child health. They are saddled with the responsibility for the health of their children and take their children for vaccination. Typically, in Somali Region of Ethiopia the power to make decisions about the immunization of children belonged to women^19^.

Another contributing factor to the high levels of SIA awareness may be the engagement and revitalization of SMCs and their broad community membership base. These committees have been widely recognized as effective in mobilizing communities to action. They possess immense potential for engaging on broader health issues. However, their functionality in general and more specifically related to response to polio outbreak has never been documented, whether in gray or published literature prior to the PCN collaborations. They were reactivated and reinvigorated to play critical roles in informing and mobilizing the people act in response to polio outbreaks in the communities.

Building on GPEI experience in other polio-endemic countries with massive vaccine resistance, which was sometimes erroneously linked to religion or politics, the collaboration with the IASC focused on a broad set of mutually developed messages, not limited to polio and was supported by Quranic teachings^13^. This strategy made it possible for the communities to reap broad health education benefits, beyond polio, and it helped to develop their confidence in the messages coming from trusted religious leaders, backed by religious teachings. These efforts were deliberate strategies, aimed to thwart any skepticism, misconceptions, fear, resentment, or resistance that a vertical, “polio-only” approach might indirectly foster.

Perhaps the most complex and most significant area of impact was the increased communication capacity and social data for the polio outbreak response programme. The PCN generated health communication capacity at various levels within the outbreak countries in the HoA. Literature show that availability of technical assistance and capacity on the ground were integral elements to the success of communication programmes^20^. Regional, zonal, and local health communication capacities within the health system structures were limited, often with no dedicated staff to support health communication and social mobilization. The PCN contributed greatly to filling this gap; placing dedicated communication staff in strategic places and built capacities of existing health staff and community members through participation in trainings, review meetings, and formal partnerships and involvement in programme implementation, monitoring, and evaluation. Initial results from a recently conducted monitoring assessment of the PCN in 2016 shows a very positive impact that the network has made in all areas supported, with extended benefits into the broader child health agenda^13^.

Enhanced understanding of the behavioral and environmental factors that affect household and community decisions to accept or seek polio and routine immunization in the region were made possible through the emphasis PCN placed on knowledge generation, quality research, monitoring, and evaluation, which helped to generate a wealth of social data. For instance, the study on zero-dose case provided information to better understand why some children miss out on polio or routine immunization and how communication could address this troubling gap. The clan and livestock assessments helped guide interventions to better identify and reach communities with information and to deliver targeted messages to mobile and nomadic populations with a cost-effective, culturally acceptable and efficient strategy. All these cumulatively resulted in improved capacities and knowledge base for polio, which enhance the potential to further utilize identified opportunities and support other initiatives “beyond polio”.

Though some challenges were initially encountered with the collection and use of social data for impact, through developed capacity, routine PCN reviews, assessments and trainings, systematic data review and analysis, and strong technical assistance, the PCN has generated a system of monitoring and evaluating communication interventions and impact^13^. There however remained a need to build on achievements of the PCN for polio, routine immunization, and child health programs. This will require significant investment in the PCN in terms of time and resources, which would allow the PCN to operate in challenging circumstances, related to security, access, and infrastructure.

All the same this documentation was faced with some limitation such as use of self-reported data, like the ZCC monthly reports and IASC activity reports, which may introduce bias. Also, the review was limited to the PCN, while there are polio partners who also contribute to communication and social mobilization for polio such as Core Group and others. Nonetheless, the findings of this paper remain valid based on the numerous sources of information, such as the SIA data, regular reports, and special research, which corroborate the main findings of the results. The successfully reviewed PCN strategies implemented during the polio outbreak response that may provide key lessons for other outbreak response initiatives.

In conclusion, the communication response to the polio outbreak in the HoA has yielded positive impact at the community and household levels. The implementation of a strategic, context-specific, multipronged approach, led to more children effectively reached with necessary health interventions. The PCN generated enormous experience and knowledge base, which can contribute to better understanding of the factors affecting the demand for and uptake of health services in the HoA countries, not just the outbreak countries. The successful establishment and operation of the PCN in response to polio outbreak in the HoA provide important lessons on how effective communication can advance the equity agenda in its efforts to reach all populations, particularly the most vulnerable, social and geographical isolated remote, or traditionally missed, with equitable health information and services for all. This has great implication for universal health coverage and vision 2030 agenda hinged on the philosophy, “live no one behind”^21–23^. Finally, this paper provides evidence to the effect that PCN impacted positively on for the polio outbreak response in the HoA and can be equally useful in future to support outbreak response elsewhere as well as another child survival programmes.
